# Analysis of Vibration Characteristics of Bridge Structures under Seismic Excitation

**DOI:** 10.3390/e26060465

**Published:** 2024-05-29

**Authors:** Ling’ai Li, Shengxiang Huang

**Affiliations:** School of Geodesy and Geomatics, Wuhan University, Wuhan 430072, China

**Keywords:** seismic wave, vibration response, time-frequency analysis, singular value decomposition, information entropy

## Abstract

Bridges may undergo structural vibration responses when exposed to seismic waves. An analysis of structural vibration characteristics is essential for evaluating the safety and stability of a bridge. In this paper, a signal time-frequency feature extraction method (NTFT-ESVD) integrating standard time-frequency transformation, singular value decomposition, and information entropy is proposed to analyze the vibration characteristics of structures under seismic excitation. First, the experiment simulates the response signal of the structure when exposed to seismic waves. The results of the time-frequency analysis indicate a maximum relative error of only 1% in frequency detection, and the maximum relative errors in amplitude and time parameters are 5.9% and 6%, respectively. These simulation results demonstrate the reliability of the NTFT-ESVD method in extracting the time-frequency characteristics of the signal and its suitability for analyzing the seismic response of the structure. Then, a real seismic wave event of the Su-Tong Yangtze River Bridge during the Hengchun earthquake in Taiwan (2006) is analyzed. The results show that the seismic waves only have a short-term impact on the bridge, with the maximum amplitude of the vibration response no greater than 1 cm, and the maximum vibration frequency no greater than 0.2 Hz in the three-dimensional direction, indicating that the earthquake in Hengchun will not have any serious impact on the stability and security of the Su-Tong Yangtze River Bridge. Additionally, the reliability of determining the arrival time of seismic waves by extracting the time-frequency information from structural vibration response signals is validated by comparing it with results from seismic stations (SSE/WHN/QZN) at similar epicenter distances published by the USGS. The results of the case study show that the combination of dynamic GNSS monitoring technology and time-frequency analysis can be used to analyze the impact of seismic waves on the bridge, which is of great help to the manager in assessing structural seismic damage.

## 1. Introduction

An earthquake results from the movement and collision of tectonic plates in the Earth’s crust. Previously, studies have been primarily based on the instantaneous dynamic and static displacement data of the inter-epicenter surface obtained from GPS observation stations, which are published by seismic research institutions (e.g., USGS, CSN). These studies are mainly used for the inversion of seismic source parameters, rapid acquisition of earthquake magnitude information, and obtaining the arrival time of the seismic waves [1,2,3,4,5,6]. However, these data are rarely applied to analyze the seismic damage of a specific bridge.

Bridge dynamic monitoring data are utilized to analyze its vibration characteristics under diverse environmental excitations. Previous research has been conducted on this topic. For instance, Huang et al. analyzed the displacement deformation and modal changes of the main girder structure of the Su-Tong Yangtze River Bridge when it encountered different environmental excitations, such as abnormal weather and traffic load during construction, based on the GPS dynamic monitoring system [7]. Magalhaes et al. utilized accelerometers to establish a dynamic monitoring system for a concrete arch bridge in Bordeaux, Portugal. They analyzed the effects of environmental factors and traffic loads on the structural modal parameters using observation time series for two consecutive years [8]. Ye et al. and Petersen et al. extracted the vibration characteristics of arch bridges and suspension bridges under wind loads based on the bridge dynamic monitoring system data [9,10]. Concerning seismic damage research on bridge structures, Siringoringo et al. analyzed the vibration response characteristics of bridge structures induced by seismic waves of different magnitudes, based on long-term observed displacement data from multiple sets of accelerometers deployed by the Hakucho Suspension Bridge health monitoring system [11]. Zhao analyzed the vibration modes of the Su-Tong Yangtze River Bridge during the Wenchuan earthquake using fast Fourier transform and finite element modeling methods [12]. It is evident that the seismic response of the bridge reflects the impact of seismic waves.

Accurately obtaining and analyzing bridge seismic response data is helpful for assessing bridge seismic damage. Time-frequency analysis methods, including Fourier transform, Wavelet transform, Hilbert–Huang transform, and their derivative algorithms, are commonly used to extract and analyze the characteristics of nonlinear and nonstationary bridge dynamic monitoring data. These methods have been extensively studied and applied in various research studies [13,14,15,16,17,18]. However, these methods have some drawbacks, including mode aliasing, endpoint effects, and difficulty extracting weak signals in the presence of strong noise interference [19,20,21]. The normal time-frequency transform (NTFT) method is based on dimensional conservation [22]. It can precisely extract the instantaneous amplitude, phase, and frequency of each component in a signal from the NTFT spectrum coefficients, and also reveal the time-varying characteristics of this information. The singular value decomposition (SVD) method decomposes a noisy signal into sub-signals, enabling the direct extraction of useful information or the combination of components to form a valid signal. SVD-based signal separation using the Hankel matrix method is commonly used for signal denoising. The resulting denoised signal does not have a phase shift when compared to the original signal [23,24,25]. Information entropy captures the uncertainty of information and is also used to measure the complexity of a system. Studies have shown that the information entropy of mechanical friction vibration signals is closely related to the change of friction coefficient. This relationship can be used to monitor and evaluate the running-in process and wear state of machinery [26,27]. Ying et al. proposed a rolling bearing fault diagnosis algorithm that utilizes ensemble entropy, the Holder coefficient theory, and the grey relation algorithm. This algorithm is suitable for online health status monitoring [28]. For a structural vibration response analysis, Zhang et al. determined the optimal length for the vibration data analysis (ODAL) based on the sensitivity of the improved multi-scale permutation entropy (IMPE) to data length [29]. Chen et al. evaluated the randomness and complexity of the noise in the vibration signal using MPE. They then denoised the signal using variational mode decomposition (VMD) and wavelet methods [30]. In light of this, the purpose of this paper is to attempt to integrate the advantages of NTFT, SVD, and information entropy in signal processing, and propose an optimized time-frequency analysis method (NTFT-ESVD) to obtain the time-frequency information of structural vibration response signals. This method has the advantage of effectively extracting useful signals from those with a limited signal-to-noise ratio. It improves the accuracy of identifying the time-frequency characteristics of the seismic response signals of bridge structures and enhances the reliability of seismic damage assessment for bridges.

The paper is organized as follows: Section 2 presents a general overview of the basic theory of data processing methods, procedures, and accuracy metrics. Section 3 describes the simulation experiments and analyzes the reliability of the NTFT-ESVD method. In Section 4, the NTFT-ESVD method is applied to extract and analyze the time-frequency characteristics of bridge vibration response signals under seismic excitation. Section 5 provides conclusions.

## 2. Methodology and Accuracy Metrics

### 2.1. The Theory of the ‘Inaction Method’

The ‘Inaction method’ is based on the normal time-frequency transform (NTFT) theory. A detailed definition of the ‘Inaction method’ can be found in the previous research [31,32,33]. A harmonic signal can be expressed as follows:

(1) $$f(t)=\left|A\right|\exp(i(\beta t+\varphi ))$$

where $\left|A\right|$ denotes the amplitude; β denotes the instantaneous angular frequency; φ denotes the initial phase; and βt+φ denotes the instantaneous phase.

The NTFT of harmonic signal f(t) is defined as follows:

(2) $$\Psi f(\tau ,\varpi )=\int_{R}f(t)\bar{\psi }(t-\tau ,\varpi )d_{t},\tau ,\varpi \in R$$

where ψ(t−τ,ϖ) denotes the kernel function; ‘–’ means taking the conjugate; τ,ϖ represent the time indices and frequency indices, respectively.

The typical NTFT kernel function can be constructed as follows:

(3) $$\psi (t,\varpi )=\left|\mu (\varpi )\right|w(\mu (\varpi )t)\exp(i\varpi t),w(t)\in \Omega (R),(\mu (\varpi )\in R)\overset{•}{\neq }0$$

where μ(ϖ) represents the time-frequency resolution adaptor (TFRA), which can be designed as any number or expression except zero; the dot above the inequality sign means ‘almost anywhere’; w(t) denotes the normal window function. The Fourier transform of the normal window function satisfies the following:

(4) $$\overset{⌢}{w}(\omega )=\max\left|\overset{⌢}{w}(\omega )\right|=1\Leftrightarrow \omega =0$$

Equation (4) illustrates that the maximum of the normal window function is obtained only when the frequency index is equal to zero.

Any NTFT to f(t) holds [32]:

(5) $$\Psi f(\tau ,\varpi )=f(\tau )\overset{⌢}{w}\left(\frac{\varpi -\beta }{\mu (\varpi )}\right)$$

where $\overset{⌢}{w}\left(\frac{\varpi -\beta }{\mu (\varpi )}\right)$ is defined as HAW (harmonic amplitude weight), which can be used to design parameters of NTFT [31].

The property of the ‘Inaction method’ to a harmonic signal is concluded as follows:

(6) $$\left|\Psi f(\tau ,\varpi )\right|=\max imum=\left|f(t)\right|\Leftrightarrow \varpi =\beta ,\forall \tau \in R$$

(7) Ψf(τ,β)=f(τ),∀τ∈R

where ∀ means ‘for any’; Equation (6) means that the value $\left|\Psi f(\tau ,\varpi )\right|$ of a harmonic signal f(t) with frequency β could reach the local maximum at any point in time τ and only if the variable ϖ is equal to β, so we need to first determine ϖ; Equation (7) indicates that the local maximum can be regarded as the instantaneous amplitude when ϖ equals β, i.e., $\left|\Psi f(\tau ,\varpi )\right|$ is exactly the harmonic signal itself. For another time point τ, repeating the steps yields the instantaneous frequency, phase, and amplitude [32]. This extraction method is called the ‘Inaction method’.

It is noteworthy that the data collected by GPS technology were a real signal. Based on ‘Euler’s formula’ ($\exp^{i\theta }=\cos\theta +i\sin\theta$), the real signal can be reconstructed according to the relationship:

(8) 2ReΨf(τ,β)=f(τ)=Acos(βτ+φ)

where ‘Re’ denotes extraction of the real part of the complex signal; the ‘Inaction method’ can be regarded as a ‘line-pass’ filter, which is suitable to extract the instantaneous narrow-band signal without inverse transformation. In contrast to the high/low-pass filters, which allow signals above or below a cutoff frequency to pass through, or the band-pass filter, which transmits a range of frequencies while excluding frequencies outside that range, a ’line-pass’ filter transmits a signal of a specific frequency, rather than a signal within a specific frequency range. For multi-component harmonic signals, the ‘Inaction method’ enables the extraction of each signal component. More details can be found in previous research [22,31].

### 2.2. Singular Value Decomposition (SVD)

Assuming that the time series of the observed data is represented by $X=(x_{1},x_{2},\cdots ,x_{N})$, it is extended to form a trajectory matrix. The trajectory matrix is selected as the Hankel matrix [34]. The length of the sliding window is set to L=N/2 during the singular value decomposition. The window is then embedded in the time series x bit by bit to form a trajectory matrix composed of (N/2+1) vectors. The expression is as follows:

(9) $$X_{m\times n}=\left[\begin{array}{cccc} x_{1} & x_{2} & \cdots & x_{N/2}\\ x_{2} & x_{3} & \cdots & x_{N/2+1}\\ \vdots & \vdots & \vdots & \vdots \\ x_{N/2+1} & x_{N/2+2} & \cdots & x_{N} \end{array}\right]$$

(10) $$x_{i}=x_{s}+x_{z},i=1,2,\cdots ,N$$

where $x_{s}$ represents the desired signal and $x_{z}$ represents the noise component of the signal.

The singular value decomposition of matrix $X_{m\times n}$ is used with the following expression:

(11) $$X_{m\times n}=U_{m\times m}\times \Sigma_{m\times n}\times {V_{n\times n}}^{T}$$

where $U_{m\times m}$ and $V_{n\times n}$ are orthogonal matrices, which satisfy $U^{T}U=I$, $V^{T}V=I$. $\Sigma_{m\times n}$ is a diagonal matrix, and its general expression is as follows:

(12) $$\Sigma_{m\times n}=\left[\begin{array}{cccc} \gamma_{1} & 0 & \cdots & 0\\ 0 & \gamma_{2} & \cdots & 0\\ \vdots & \vdots & \vdots & \vdots \\ 0 & 0 & \cdots & \gamma_{m} \end{array}\right]$$

**Figure 1:**
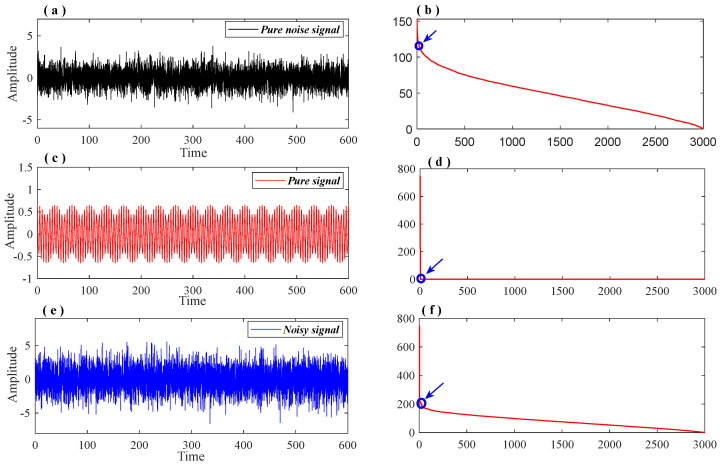
The singular value distribution curve of the signal. (**a**,**c**,**e**): The pure noise signal, pure signal and noisy signal; (**b**,**d**,**f**): Singular value distribution curves of the signals corresponding to sub-figures (**a**,**c**,**e**) respectively.

**Figure 2:**
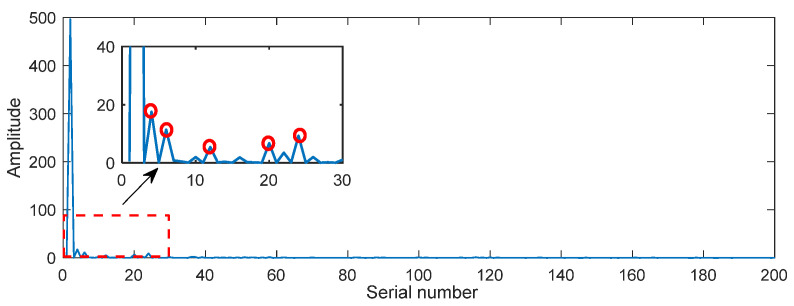
Singular value difference spectrum of the noisy signal.

The diagonal element $\gamma_{1},\gamma_{2},\cdots ,\gamma_{m}$ represents the singular value of the matrix $X_{m\times n}$. Figure 1 displays the distribution of singular values of signals with varying noise levels. It is evident that the slope convergence of singular value distribution curves differs for different signal types. Additionally, a singular value difference spectrum is employed and defined as $\delta_{i}=\left|\gamma_{i+1}-\gamma_{i}\right|$. For signal reconstruction, the first k (k << m) singular value is retained and the remaining singular values are zeroed. The signal is then reconstructed using Equation (13) and the anti-angle element in X(S) is averaged to obtain the denoised useful signal [35,36]. The number of valid singular values k is usually determined by the maximum peak of the singular value difference spectrum. However, in some cases, excessive denoising can lead to an incomplete extraction of the useful signal. In such cases, suspicious peaks are identified as ‘ambiguity points’ based on the singular value curve of the signal and the peaks distribution of the difference spectrum, as shown in Figure 2. The singular values corresponding to all ‘ambiguity points’ are used to reconstruct the signal, and the most effective reconstruction is determined using the information entropy index.

(13) $$X(S)=U\Sigma_{k}V^{T}=U\left[\begin{array}{cc} \Sigma_{k} & 0\\ 0 & 0 \end{array}\right]V^{T}$$

### 2.3. Information Entropy

Information entropy is a fundamental concept in information theory. It was first introduced by Claude Shannon in his paper, ‘A Mathematical Theory of Communication’, in 1948 to address the challenge of quantifying information. When calculating information entropy, it is necessary first to determine all possible states in a dynamic system. Then, one must calculate the probabilities of each state and use them to calculate the information entropy. The formula for information entropy is as follows [27,28]:

(14) $$H(X)=-\sum_{i=1}^{n}p(x_{i})\log p(x_{i})$$

where $p(x_{i})$ represents the probability of X being $x_{i}$. Equation (14) demonstrates that the information entropy of a system is inversely proportional to the probability of each state multiplied by the logarithmic sum of the probabilities. A high information entropy indicates high uncertainty or complexity in a dynamic system. Conversely, a lower information entropy indicates a simpler system structure with fewer types of situations.

The information entropy H(X) of the discrete random variable X represents the mathematical expectation of the amount of information. The greater the entropy, the higher the amount of information in X, and the higher the uncertainty. Conversely, the smaller the entropy, the higher the certainty, and the fewer components in X.

The vibration response signal of the bridge can be viewed as a collection of discrete random variables. To extract a single signal component, the ‘Inaction method’ is used to process the vibration signal. Noise, on the other hand, is irregular and disordered in comparison to a single and definite signal. The randomness characteristic of noise renders it more uncertain and complex. According to the property of information entropy, it can be determined that the information entropy of noise is higher than that of a pure signal. Consequently, when noise is mixed into the signal, the certainty of the original signal is reduced, and the information entropy of the signal is increased. Therefore, information entropy can be used to select the most effective SVD denoising reconstruction signals. The specific steps are as follows:

(1) The singular values corresponding to the ‘ambiguity points’ determined in the signal singular value difference spectrum shown in Figure 2 are selected for signal reconstruction.
(2) The information entropy is calculated for each signal reconstructed in step (1).
(3) The reconstruction signal with the smallest information entropy value in step (2) is determined as the most effective SVD reconstruction signal.

### 2.4. Data Processing Procedures

**Figure 3:**
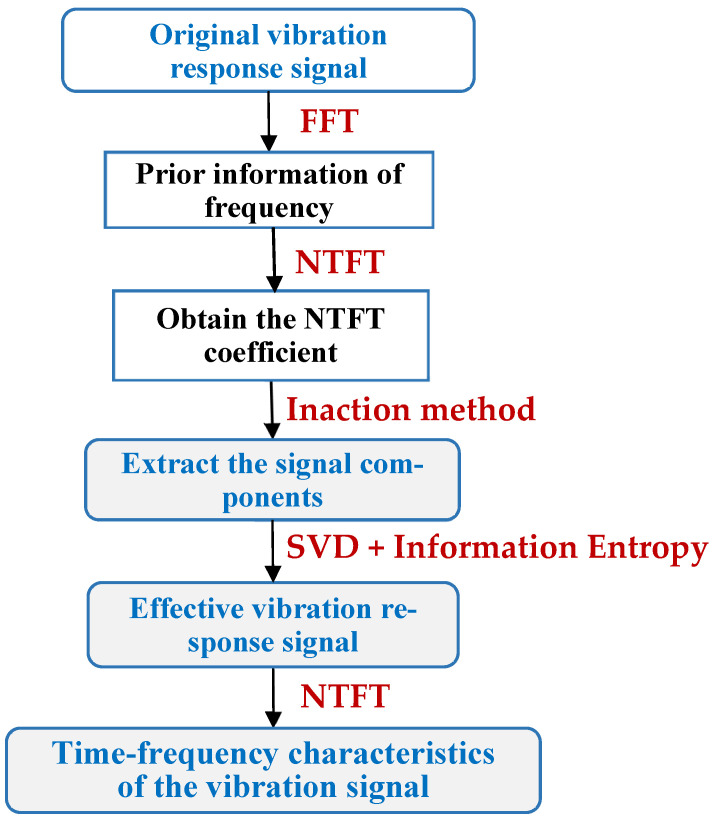
The analysis flowchart of the vibration response signal.

Figure 3 shows the data process flow of extracting time-frequency information from the vibration response signal. First, the FFT method is used to obtain the prior frequency information of the original signal. The signal components are then extracted using the ‘Inaction method’. Next, the information entropy-based SVD method is used to denoise the extracted signal components, resulting in an effective vibration response signal. Finally, the time-frequency characteristics of the vibration response signal can be determined by using the NTFT method again.

### 2.5. Accuracy Evaluation Metrics

The relative error of the calculation result refers to the ratio of the absolute error to the true value multiplied by 100%, expressed as a percentage. In general, the relative error can better reflect the reliability of the calculation results. The relative error can be expressed as follows:

(15) $$\delta =\left|\left(l-L\right)\right|/L\times 100\%$$

where l represents the calculation result; L represents the true value.

The root mean square error (RMSE) is used to measure the deviation between the calculated value and the true value, which is sensitive to outliers in the data. The Pearson correlation coefficient (r) is a widely used measure of the degree of correlation between two variables with a value between −1 and 1. Their expression is as follows:

(16) $$RMSE=\sqrt{\frac{1}{N}\sum_{i=1}^{n}{\left(Y_{i}-X_{i}\right)}^{2}}$$

(17) $$r=\frac{\sum_{i=1}^{n}\left(X_{i}-\bar{X})(Y_{i}-\bar{Y}\right)}{\sqrt{\sum_{i=1}^{n}{\left(X_{i}-\bar{X}\right)}^{2}}\sqrt{\sum_{i=1}^{n}(Y_{i}-\bar{Y}{)}^{2}}}$$

where $Y_{i}$ and $X_{i}$ denote the calculation results and the true value, respectively.

The signal-to-noise ratio (SNR) is a crucial indicator of signal quality. It measures the ratio between the strength or power of the signal and the noise. A higher SNR indicates a stronger signal relative to noise and better quality. The expression for the SNR is as follows.

(18) $$SNR=10*\mathrm{lg}(\frac{P_{s}}{P_{n}})$$

(19) $$P_{s}=\frac{\sum_{i=1}^{n}{X_{i}}^{2}}{N}$$

(20) $$P_{n}=\frac{\sum_{i=1}^{n}{\left(Y_{i}-X_{i}\right)}^{2}}{N}$$

where $P_{s}$ stands for signal power and $P_{n}$ stands for noise power.

## 3. Simulation Experiment

### 3.1. Introduction to the Simulation Experiment

**Figure 4:**
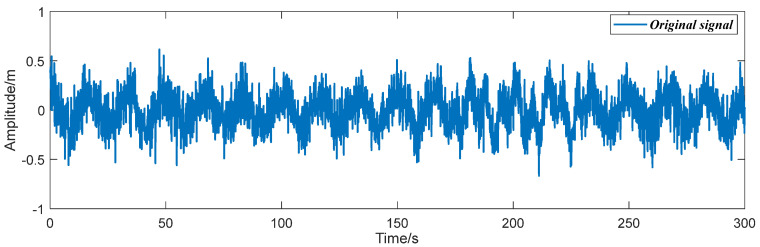
The simulated signal with a signal-to-noise ratio of −2.

Following an earthquake, the bridge structure is subjected to the influence of seismic waves [37]. Analog signals are conducted to simulate the vibration response of the bridge structure when exposed to seismic waves. The signal comprises two components: signal S1, simulating the gentle vibration of the structure without extreme environment excitation; and signal S2, simulating the seismic response of the structure. A continuous, aperiodic square wave pulse with a height of one is added to signal S2. The pulse is centered at t=200s and has a width of 80. Signal 2 simulates the vibration response for 80 s, reaching its maximum amplitude at t=200s. Gaussian white noise with a signal-to-noise ratio of −2 is incorporated into the simulated signal to represent ambient noise. The mathematical representation of the simulated signal is as follows:

(21) $$f(t)=\left\{\begin{array}{c} A_{1}\cos(2\pi f_{1}t)+noise,1\leq t\leq 3000\\ \left[A_{2}\sin(2\pi f_{2}t)*\sin(2\pi f_{3}(t+7))\right]+noise,1600\leq t\leq 2400 \end{array}\right.$$

where the amplitudes of two signal components are set as $A_{1}=0.15\mathrm{mm}$ and $A_{2}=0.2\mathrm{mm}$, respectively. The frequencies of the simulated signal are separately designed as $f_{1}=0.06\mathrm{Hz}$, $f_{2}=0.15\mathrm{Hz}$, and the modulated signal frequency $f_{3}=0.006\mathrm{Hz}$. The sampling frequency $f_{s}$ of the simulated signal is set as 10 Hz with 3000 sampling points and the sampling interval $T_{s}$ is 0.1 s. The simulated signal is shown in Figure 4.

### 3.2. Results and Discussion

**Figure 5:**
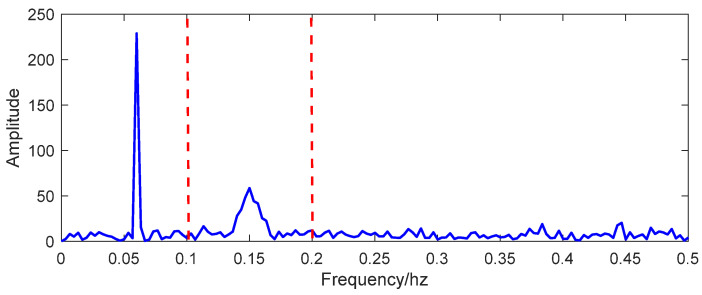
The FFT spectrum of the simulated signal.

**Figure 6:**
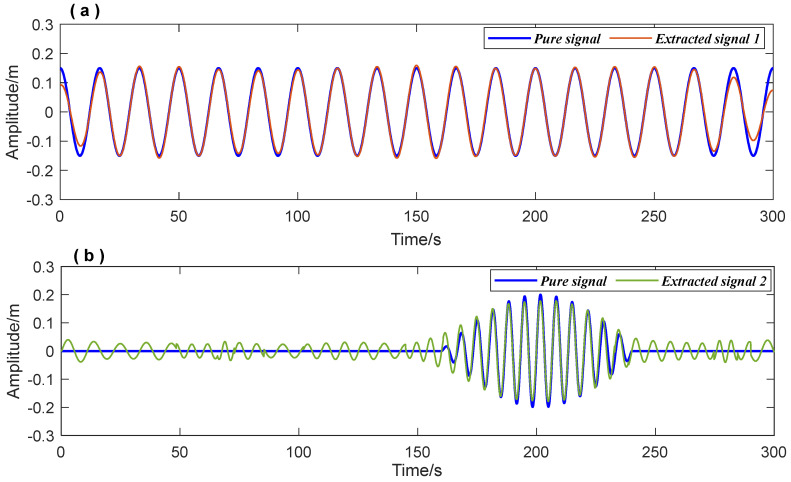
The components’ extraction results of simulated signal by the ‘Inaction method’ (**a**) Extraction results of Signal 1; (**b**) Extraction results of Signal 2.

**Figure 7:**
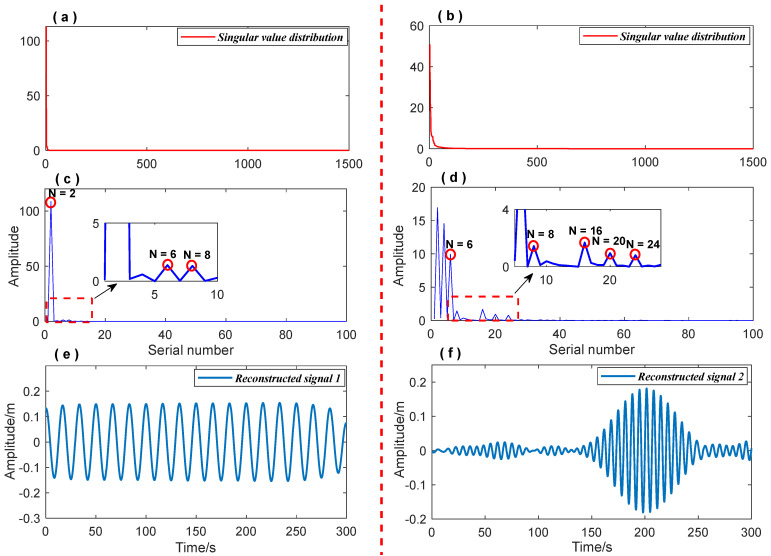
The signal extracted by NTFT was processed using the SVD method. (**a**,**b**): Singular value distribution curves of the Signal 1 and Signal 2; (**c**,**d**): The ‘ambiguity points’ distribution of singular value difference spectrum; (**e**,**f**): Extraction results of Signal 1 and Signal 2 using NTFT-ESVD method.

**Figure 8:**
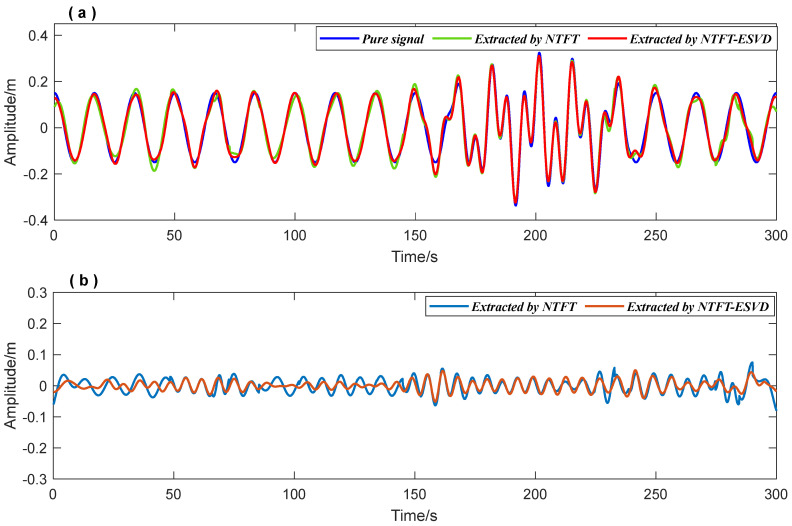
The results of the signal extraction using the NTFT method and the NTFT-ESVD method. (**a**) Extraction results of the simulation signal; (**b**) Deviation statistics of signal extraction results.

**Figure 9:**
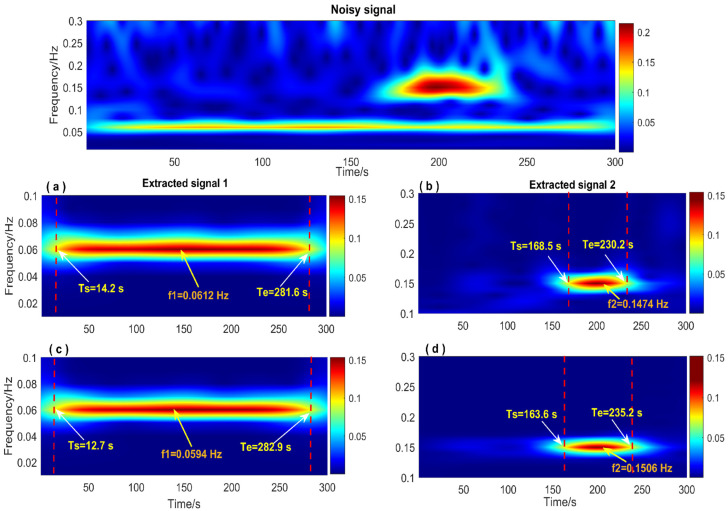
The time-frequency spectrum of the simulated signal. (**a**,**c**): Signal 1 extracted by the NTFT method and the NTFT-ESVD method; (**b**,**d**): Signal 2 extracted by the NTFT method and the NTFT-ESVD method.

Accurately extracting signal components can improve signal time-frequency feature recognition precision. The data processing procedure is shown in detail in Figure 3. First, the FFT method was used to obtain prior frequency information of the simulated vibration signal. As shown in Figure 5, the spectrum illustrated two frequency ranges of the signal components: [0.01 Hz 0.1 Hz] and [0.1 Hz 0.2 Hz]. The ‘Inaction method’ was then employed to extract signal S1 and signal S2. Figure 6 shows the extraction results, which revealed significant distortion, especially for signal component 2 (Figure 6b). To enhance the accuracy of signal extraction, we applied the SVD method to denoise each signal component extracted using the ‘Inaction method’. Figure 7 shows the ‘ambiguity points’ of the singular value difference spectrum of the two signal components were determined according to the singular value distribution curves (Figure 7a,b) and the peak distribution of the difference spectrum (Figure 7c,d), respectively. Table 1 shows the statistical results. For signal 1, the information entropy of the reconstruction result had the smallest value (3.5294) when the number of singular values was two. Similarly, for signal 2, the information entropy of the reconstruction result had the smallest value (2.6903) when the number of singular values was six. Based on the theoretical analysis conclusion of information entropy in Section 2.3, the most effective SVD denoising signal was determined as the reconstruction result with the minimum information entropy of each component, as depicted in Figure 7d,e. Figure 8a compares the results of direct signal extraction using the ‘Inaction method’ and the results of further signal extraction using the SVD method based on information entropy evaluation metrics, and it can be seen that the signal extracted using the NTFT-ESVD method is closer to the pure signal. The deviation of the two signal extraction methods shown in Figure 8b further demonstrates that the signal extracted using the NTFT-ESVD method was more accurate. Table 2 provides detailed statistics on the performance of the signal extraction method (NTFT-ESVD). The signal-to-noise ratio was significantly improved from −2 to 17.4611, the correlation coefficient was increased from 0.6094 to 0.9910, and the root mean square error was reduced from 0.1492 to 0.0159. It can be concluded that each signal component extracted using the ‘Inaction method’ required additional noise reduction in conjunction with the SVD method. Finally, the NTFT method was applied to analyze the time-frequency information of the two signal components. Figure 9 shows the time-frequency characteristics of the signal components extracted using the two methods.

Figure 9a,b show the time-frequency analysis results of the signal extracted directly using the NTFT method. Figure 9c,d show the time-frequency feature recognition results of the signal extracted using the NTFT-ESVD method. Table 3 compares the time-frequency feature recognition accuracy of the signals extracted using the two methods. It can be seen that the accuracy of the time-frequency analysis of the signal extracted by the NTFT-ESVD method was significantly better than that of the NTFT method, especially since the time-frequency parameter recognition result of signal 2 was more obvious. The maximum relative error of the signal components extracted by the NTFT-ESVD method was only 1%, and the maximum relative error of the time parameter detection result was not more than 6%. The aforementioned simulation results demonstrate the feasibility and reliability of the NTFT-ESVD method in extracting structural seismic response signals. In addition, the time-frequency characteristics of the seismic response signal for the structure aided in evaluating the impact of seismic waves on the safety of the structure.

It is worth noting that the NTFT-ESVD method was not optimal for extracting weak signals submerged in strong noise, such as intermittent vibration response signals with signal-to-noise ratios of less than −10. This difficulty arose because it was challenging to accurately obtain prior frequency information of weak signals from the FFT spectrum for filtering.

## 4. A Case Study of the Su-Tong Yangtze River Bridge

### 4.1. The Seismic Event and Data Sources

#### 4.1.1. Seismic Event

**Figure 10:**
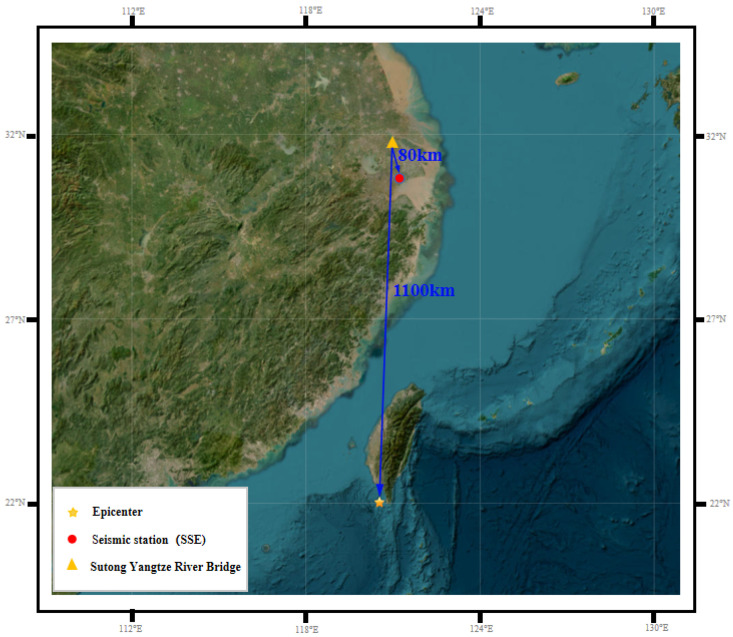
Overview map showing the location of the Hengchun earthquake, the Su-Tong Yangtze River Bridge, and the seismic station.

On 26 December 2006, a magnitude 7.1 earthquake occurred in the Luzon Strait at 12:26:21.0 UTC. The epicenter was 22.89 km from the Hengchun Seismological Station in Pingtung County, Taiwan Province, China. The epicenter of the earthquake was located at 21.69° N, 120.56° E at a depth of 44.1 km. The relationship between the epicenter of the Hengchun earthquake and the location of the Su-Tong Yangtze River Bridge is depicted in Figure 10. The yellow asterisk marks the location of the epicenter, the red dot marks the Shanghai Seismic Station, and the yellow triangle marks the location of the Su-Tong Yangtze River Bridge. Given that the distance between the epicenter and the Su-Tong Yangtze River Bridge is approximately 1100 km, the bridge was affected by teleseismic waves.

#### 4.1.2. Data Source

**Figure 11:**
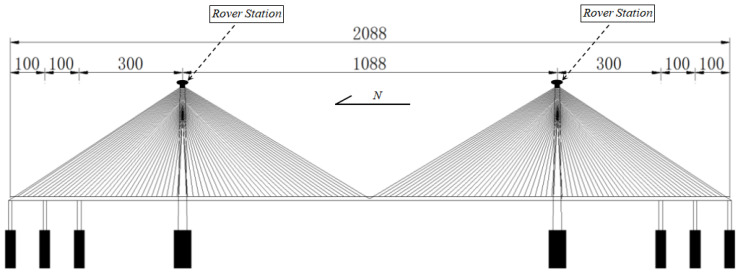
The GPS receivers were arranged at the top of the bridge tower.

The Su-Tong Yangtze River Bridge in China’s Jiangsu Province is a significant cable-stayed bridge with a total length of 2088 m. The main span of the bridge is 1088 m, and the north and south pylons are each 300.4 m height. During the construction of the bridge, a dynamic GPS real-time monitoring system was installed to monitor the deformation of the structure, and the Trimble 4700 GPS receivers were used to collect the structural dynamic monitoring data. A GPS receiver was placed as a reference station on a stable structure near the end of the bridge, while two GPS receivers were placed separately on the north and south cable-stayed pylons as rover stations (shown in Figure 11). The sampling rate of all GPS receivers was set to 10 Hz.

**Figure 12:**
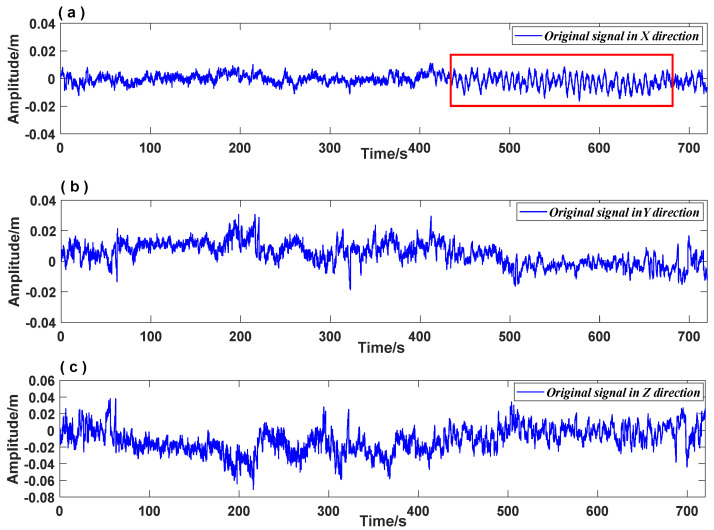
The vibration response signal in three dimensions. (**a**): The vibration response signal in the X direction; The red rectangular box shows the obviously abnormal vibration; (**b**): The vibration response signal in the Y direction; (**c**): The vibration response signal in the Z direction.

**Figure 13:**
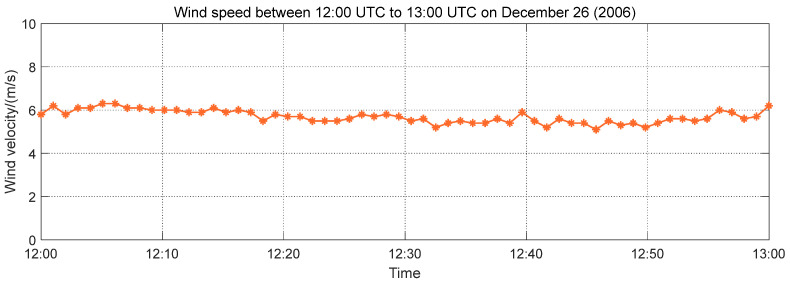
The wind speed measured from 12:00 UTC to 13:00 UTC by the anemometer.

In this study, the vibration response signals of the GPS real-time dynamic monitoring system of the bridge in three dimensions during the Hengchun earthquake were selected as research objects, as shown in Figure 12. The vibration response signals were collected from 12:26:00 UTC to 12:37:00 UTC on 26 December 2006, lasting for 12 min. The X direction indicates the direction along the bridge (Figure 12a), the Y direction indicates the transverse direction of the bridge (Figure 12b), and the Z direction indicates the vertical direction of the bridge (Figure 12c). The wind speed measured by the anemometer from 12:00:00 UTC to 13:00:00 UTC on 26 December 2006 is shown in Figure 13. The wind load during the earthquake was a moderate breeze, which can be excluded from wind-related abnormal vibrations of the bridge structure. Therefore, the vibration response signal in this period was utilized to analyze the effects of seismic waves on the Su-Tong Yangtze River Bridge during the Hengchun earthquake.

### 4.2. Result and Discussion

#### 4.2.1. Extracting the Main Frequency Vibration Signal

The primary frequency signal of the vibration response of the bridge in the three-dimensional direction reflects the influence of seismic waves on the bridge structure. Based on the propagation velocity of the seismic waves, we can assume that the P wave and S wave mainly caused the vibration response in the Z direction (Figure 12c) and Y direction (Figure 12b) of the bridge. The vibration response in the X direction, marked by a red rectangle in Figure 12a, displaying evident harmonic motion characteristics, may be attributed to the surface wave.

**Figure 14:**
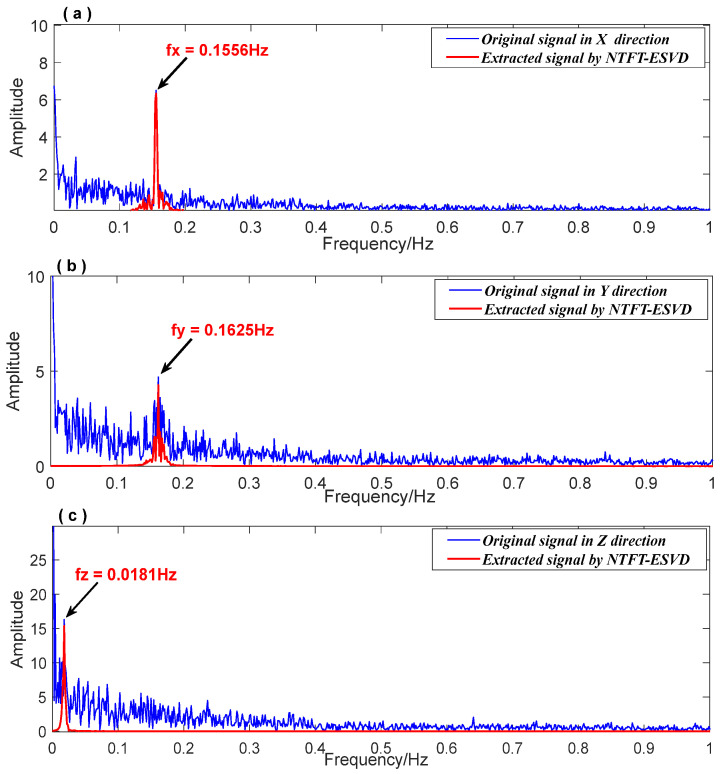
The FFT spectrum of the vibration response signal in three-dimensional direction. (**a**): The FFT spectrum in the X direction; (**b**): The FFT spectrum in the Y direction; (**c**): The FFT spectrum in the Z direction.

**Figure 15:**
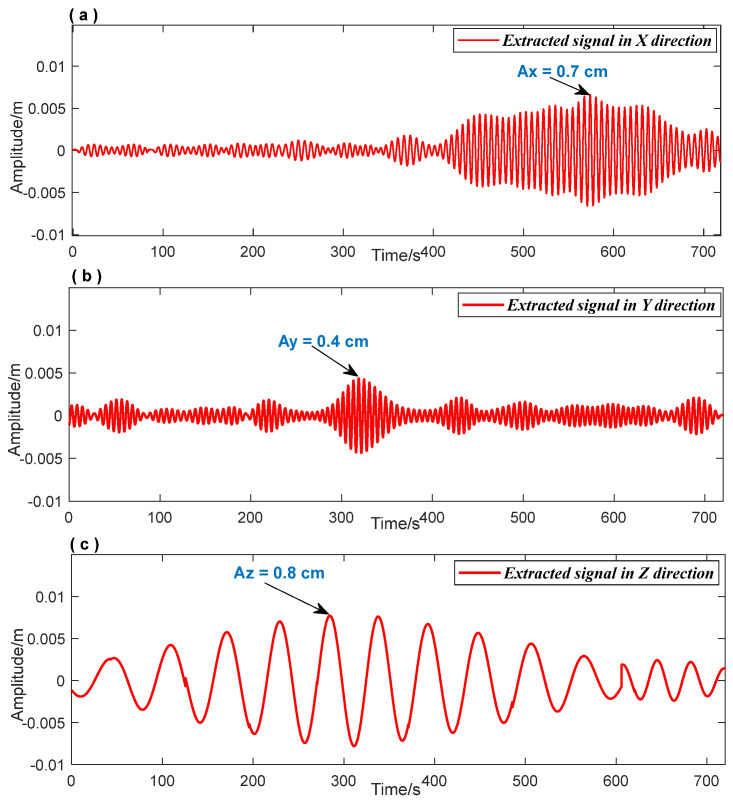
The extracted signal in three-dimensional direction. (**a**): Signal extraction results in the X direction; (**b**): Signal extraction results in the Y direction; (**c**): Signal extraction results in the Z direction.

Based on the data processing flow shown in Figure 3, the FFT method was used to obtain the prior frequencies ($f_{x}=0.1556\;\mathrm{Hz}$, $f_{y}=0.1625\;\mathrm{Hz}$, and $f_{z}=0.0181\mathrm{Hz}$) of the vibration response signals in each direction of the bridge structure. According to the FFT spectrum shown in Figure 14 (blue line), the frequency domain ranges for extracting signals were set as [0.1 Hz 0.3 Hz], [0.1 Hz 0.3 Hz], and [0.01 Hz 0.03 Hz] for the three-dimensional vibration response signals. The red line in Figure 14 indicates that the signal extracted using the NTFT-ESVD method had a low level of noise. Figure 15 shows the seismic response signal of the bridge structure in a three-dimensional direction extracted using the NTFT-ESVD method. It is evident that the maximum amplitudes of vibration in each direction did not exceed 1 cm, which were Ax=0.7 cm, Ay=0.4 cm, and Az=0.8 cm, respectively. Furthermore, the modal damping ratios of the seismic response of the bridge structure were calculated using the time-domain logarithmic attenuation method (Equation (22)) based on the main frequency vibration signals extracted from the three-dimensional directions. The resulting values were $\zeta_{x}=0.0118$, $\zeta_{y}=0.0302$, and $\zeta_{z}=0.0354$, respectively. The results of this part reveal that the teleseismic waves mainly induced the low-frequency and small-amplitude vibration response of the bridge structure:

(22) $$\zeta =\frac{1}{2\pi \cdot n}\cdot ln\frac{A_{h}-A_{l}}{A_{h}^{\prime }-A_{l}^{\prime }}$$

where n denotes the number of the waves involved in the calculation, which is not less than 3; $A_{h}$ and $A_{l}$ represent the peak and trough values of the first wave, respectively; $A_{h}^{\prime }$ and $A_{l}^{\prime }$ denote the peak and trough values of the tail wave involved in the calculation, respectively.

#### 4.2.2. The Time-Frequency Characteristics of Structural Vibration Response Signal

In Section 4.1.1, the seismic response signal of the Su-Tong Yangtze River Bridge in three-dimensional directions during the Hengchun earthquake was extracted using the NTFT-ESVD method. This section presents the time-frequency characteristics of these vibration signals obtained using the NTFT method, which will be helpful in analyzing the effects of seismic waves on the safety of bridge structures.

**Figure 16:**
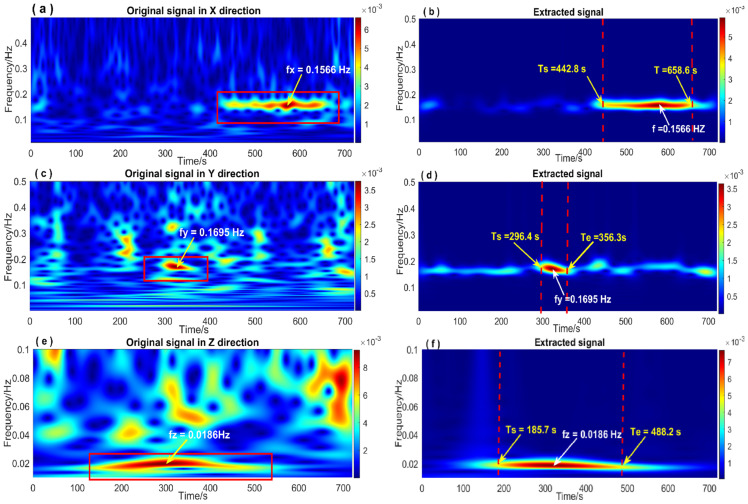
The NTFT spectrum of the extracted signal in three-dimensional direction. (**a**,**c**,**e**): Time-frequency spectrum of the original vibration response signals in the X, Y, and Z directions; (**b**,**d**,**f**): Time-frequency spectrum of the vibration response signals extracted in the X, Y, and Z directions.

In Figure 16, the time-frequency spectrum before and after signal extraction is illustrated. Clearly, the NTFT-ESVD method effectively extracted the structural vibration response signal under seismic excitation, and its NTFT time-frequency spectrum significantly facilitated the accurate acquisition of the time-frequency characteristics of the vibration signal. According to the propagation speed of the seismic wave, the vertical direction (Z direction) of the bridge structure was the first to be hit by P waves. As shown in Figure 16f, the frequency of the detected vibration response signal was *f_z_* = 0.0186 Hz. The vibration started at $T_{s}=185.7\;s$, which corresponds to 12:29:05.7 (UTC), and ended at $T_{e}=488.2\;s$, indicating that the bridge structure had about five responses to the P wave for several minutes. The S wave reached the Earth’s surface after the P wave and caused the bridge structure to vibrate horizontally. As shown in Figure 16d, the starting time of the bridge vibration response caused by the S wave was $T_{s}=296.4\;s$, which corresponds to 12:30:56.4 UTC, and ended at $T_{e}=356.3\;s$. The vibration response lasted about 1 min, and the vibration frequency of the bridge structure was *f_y_* = 0.1695 Hz. Surface waves are excitations of P and S waves when they collide on the surface. The Hengchun earthquake’s surface wave caused harmonic vibration in the axis direction of the Su-Tong Yangtze River Bridge (marked by a red rectangular box in Figure 12a). Figure 16a shows the time-frequency characteristics of this vibration response. The structure’s vibration frequency was *f_x_* = 0.1566 Hz. The vibration response started at $T_{s}=442.8\;s$ and ended at $T_{e}=658.6\;s$, indicating the harmonic vibration lasted about 4 min.

Furthermore, taking into account the propagation distance of seismic waves, the P wave and S wave arrival times of three seismic stations (SSE/WHN/QZN) with similar epicenter distances to the Su-Tong Yangtze River Bridge are introduced in this part, as published by the USGS. As shown in Table 4, the ‘ST-GPS’ represents the starting moment of the seismic response at the Su-Tong Yangtze River Bridge. The comparison results demonstrate that the combination of GPS technology and the time-frequency analysis method is a reasonable and reliable approach to obtain the time of seismic waves arriving at the structure in real time.

## 5. Conclusions

Seismic waves cause up-and-down vibrations and horizontal shaking of the ground in a certain range, thus affecting the stability and safety of buildings. In this paper, the simulated vibration response signals induced by seismic wave impacts are generated using a rectangular pulse-modulated signal, and the accuracy and reliability of the NTFT-ESVD method for extracting the effective vibration response signal are analyzed. In the NTFT-ESVD method, the ‘Inaction method’ extracts the signal based on the frequency domain range of the signal components delineated by the FFT spectrum. The signal components extracted are processed using the SVD method. The trade-off problem of the number of the best singular values is solved based on the information entropy evaluation index and the ‘ambiguity point’ in the singular value difference spectrum of the signal. The final SVD reconstruction results effectively retain the useful signal while removing the residual noise. Simulation experiments demonstrate that the SNR of the signal processed by the NTFT-ESVD method is improved from −2 to 17.4611. The correlation coefficient with the pure signal is increased by 62.6%, and the relative error of the time-frequency parameter calculations is no more than 6%. Based on the dynamic response monitoring data of the Su-Tong Yangtze River Bridge during the Hengchun earthquake, the NTFT-ESVD method is used to extract the effective vibration response signal of the bridge structure in the three-dimensional direction. The time-frequency characteristics obtained from the spectrum reveal that the seismic waves of the Hengchun earthquake mainly caused short-term vibration of the bridge structure with low frequency and low amplitude, which would not affect the safety of the bridge structure.

The results of this paper can aid decision-makers in promptly detecting and analyzing the seismic response of structures. This is significant for assessing the impact of seismic waves on structural stability and safety. It is worth noting that the Hengchun earthquake falls into the category of large earthquakes, but its epicenter distance from the Su-Tong Yangtze River Bridge was more than 1000 km, making it also classified as a teleseismic earthquake. Therefore, we can generalize the research scenario to analyze the damaging effects of nearby earthquakes as well as smaller magnitude seismic waves’ impact on building structures in the future.

## Figures and Tables

**Table 1:** Information entropy of each reconstructed signal with different SVD ‘ambiguity points’.

	Reconstructed Signal 1			Reconstructed Signal 2				
**N**	2	6	8	6	8	16	20	24
**Entropy**	3.5294	3.5805	3.5899	2.6903	2.8873	2.9752	3.1468	3.1317

‘N’ represents the number of singular values corresponding to the ‘ambiguity point’.

**Table 2:** The signal extraction performance of NTFT and NTFT-ESVD.

Method	Signal								
	Signal 1			Signal 2			Simulated Signal		
	RMSE	r	SNR	RMSE	r	SNR	RMSE	r	SNR
**Original signal**	/	/	/	/	/	/	0.1492	0.6094	−2.0000
**NTFT**	0.0126	0.9931	18.5152	0.0221	0.9161	7.5314	0.0236	0.9801	14.0228
**NTFT-ESVD**	0.0085	0.9969	21.9291	0.0152	0.9252	10.8002	0.0159	0.9910	17.4611

**Table 3:** Accuracy of extracting time-frequency parameters of the simulated signal.

Parameter	NTFT						NTFT-ESVD					
	Signal 1			Signal 2			Signal 1			Signal 2		
	True Value	Calculate	Relative Error/%	True Value	Calculate	Relative Error/%	True Value	Calculate	Relative Error/%	True Value	Calculate	Relative Error/%
**Amplitude/mm**	0.15	0.1592	6.1	0.20	0.1780	11	0.15	0.1495	0.3	0.20	0.1882	5.9
**Frequency** **/Hz**	0.06	0.0612	2.0	0.15	0.1474	1.7	0.06	0.0594	1.0	0.15	0.1506	0.4
**Beginning Time/s**	0	14.2	4.7	160	168.5	10.6	0	12.7	4.2	160	163.6	4.5
**Ending Time/s**	300	281.6	6.1	240	230.2	12.2	300	282.9	5.7	240	235.2	6.0

**Table 4:** The table compares the arrival time of P waves and S waves with seismic stations SSE, WHN, and QZN.

Station	Epicenter Distance/km	Latitude/°N	Longitude/°E	Arrival Time	
				P Wave	S Wave
**ST-GPS**	1190	31.79	121.00	**12:29:05.7**	**12:30:56.4**
**SSE**	1017	31.10	121.19	12:28:34.3	12:30:20.5
**QZN**	1154	19.03	109.84	12:28:50.3	12:30:41.3
**WHN**	1132	30.54	114.35	12:28:49.0	12:30:40.0

## Data Availability

Data available on request from the authors.
